# Socio-Economic Vulnerability to Climate Change in Rural Areas in the Context of Green Energy Development—A Study of the Great Masurian Lakes Mesoregion

**DOI:** 10.3390/ijerph20032689

**Published:** 2023-02-02

**Authors:** Katarzyna Kocur-Bera, Szymon Czyża

**Affiliations:** Faculty of Geoengineering, University of Warmia and Mazury in Olsztyn, 10-719 Olsztyn, Poland

**Keywords:** climate change, green energy, vulnerability, exposure, sensitivity, adaptive capacity

## Abstract

Green energy production has become a common and recognized method of electricity generation. Giving up reliance on non-renewable energy sources is an important trend in the economies of many countries. The paper presents an analysis of the impact of indicators like increased green energy production on the level of vulnerability to climate change. The model of the Climate Change Vulnerability Index (VCC) recommended by the Intergovernmental Panel on Climate Change (considering three aspects: exposure, vulnerability, and adaptive capacity of the studied spatial unit/society) was applied. Sensitivity analysis, spatial heterogeneity, and temporal dynamics of indicators characterizing changes in electricity consumption, renewable energy production, greenhouse gas emissions, and variability of financial losses due to extreme weather events and their number were implemented. Several findings arose. First, the vulnerability to climate change (the level of the VCC index), does not decrease after the implementation of a single action, like the development of green energy production. The level of index of vulnerability to climate change (VCC1) from the reference year (2017) relative to VCC2 (2021) has changed slightly, despite the development of RES. The variation does not exceed a 1% reduction in the value of the VCC1 index. Second, the decrease in the level of the vulnerability requires global, coordinated action. The value of the VCC3 index, reflecting, including changes in green energy production (X15), electricity consumption/inhabitant (X38), and green-house gas emissions (X14), exhibited more favorably the impact of these indicators on vulnerability to climate change, compared to the VCC1 reference value. In eleven poviats, the VCC3 index decreased between 1 and 4%. In seven of these poviats, green energy production increased, resulting in an average 10% decrease in the X15 indicator, the X14 indicator representing green-house gas emissions decreased by an average of 7%, while the X38 indicator describing electricity consumption/per capita decreased by an average of 16%. Third, harmonized and inclusive action by the population holds the potential to be the clue to reducing vulnerability to climate change

## 1. Introduction

**Figure 1:**
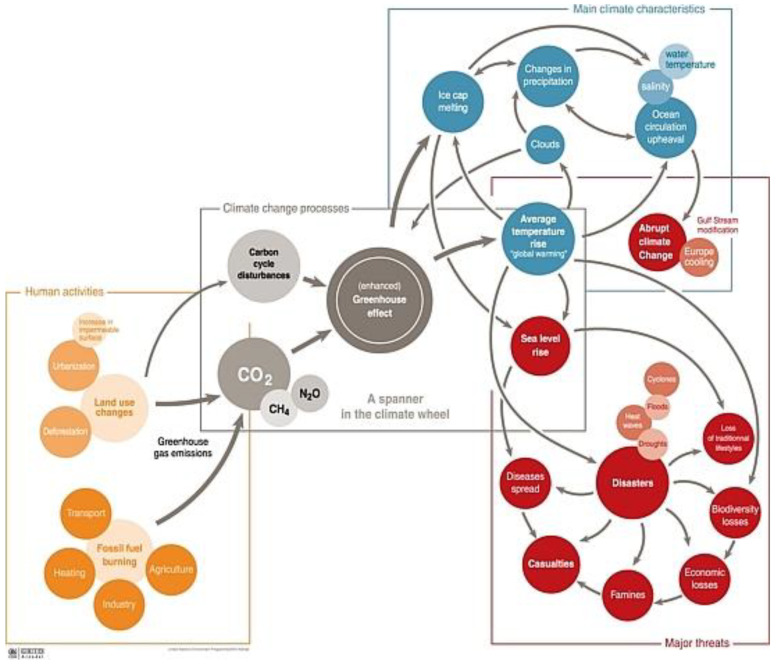
Climate change: processes, characteristics, and threats. Source: [2].

Climate change is currently one of the key environmental, social, economic, and political problems in numerous countries [1]. The recent decades have brought multiple studies of natural and social systems, confirming climate change and the impact of this variability on the environment and on human populations. This has been accepted by the authorities in the majority of countries around the world, and measures have started to be implemented aimed at minimizing the impact of human activities on space. Human activities involving changes in land use from natural or semi-natural towards urbanization and exploitation of fossil fuels (for transport, heating, industry, and agriculture) increase carbon dioxide emission to the atmosphere and carbon circulation in nature [2,3]. In the resulting greenhouse effect, the average temperature increases, the levels of seas and oceans rise, and the number and extent of natural disaster increases. Natural disasters increase the death toll, famine, disease spread, financial loss, decrease in biodiversity, etc. [2] (see Figure 1). National policies regarding climate change aim at (a) reducing the greenhouse gas emission, (b) trading in emission allowances, which helps to reduce greenhouse emissions, (c) financial support regarding climate protection, (d) reducing greenhouse gas emissions in transport, agriculture, and forestry, (e) promoting the development of low-emission technologies, (f) reducing the use of fluorinated greenhouse gases, (g) reducing the use of ozone-depleting gases and replacing them with others, harmless to the climate, (g) improving energy efficiency, (h) increasing the use of renewable energy sources [4,5]. All these measures are intended to decrease the climate change impact on society and increase the resistance to climate change.

The industrialized nations are expected to significantly reduce the consumption of fossil fuels, the largest source of human contribution to modern greenhouse gas emissions [6]. The utilization of renewable energy sources and the potential of renewable energy has recently been recognized to an intensified range. The motivation for increasing the utilization and promotion of RES includes the ability to provide for society’s energy demands as an environmentally friendly source, as well as the impact on socioeconomic vulnerability by reducing energy imports and providing reliable access to energy for citizens and industry, and improving the economic situation of development (e.g., RES create employment in the state) [4,5,6,7]. Fossil fuels are non-renewable and will be depleted [5]. Additionally, emissions from their usage are a major climate change driver [8,9,10,11]; thus, many advocate moving away from fossil fuels toward renewable energy sources (acr. RES) [12,13,14,15]. Among renewables, are wind, solar, hydro, geothermal, and bioenergy [16]. Many studies show that among RES, bioenergy is a relatively more reliable source than wind/solar because of depending on local weather conditions and seasons [17,18,19]. RES development also potentially reduces greenhouse gas emissions [20]. Many studies indicate that RES could be an attractive climate change mitigation strategy [21,22,23]. The policy in Poland targets the development of installations that produce solar energy. According to SolarPower Europe, Poland was the fourth in Europe in 2021 in terms of the installation of new photovoltaic panels [24]. Solar energy has many health as well as socioeconomic benefits for people living in rural areas [25,26,27]. The results of studies [25,26,27,28,29] revealed that the advantages of using green energy include educational benefits, provides innovative ideas for socialization and entertainment, economic benefits include financial savings on lighting, extended working hours in business, worksites, and at home, reduced emissions that additionally help maintain and improve the health of people protects against several respiratory problems in addition to environmental protection [25,26,27,28,29]. A number of these features are involved in establishing the socioeconomic vulnerability of the population living in rural areas. The deployment of green energy [29] also provides an important contribution to the Global Climate Action Plan.

The literature on the vulnerability to climate change presents two main trends in climate change analyses. The first takes into consideration only the weather-related factors and their changes [30,31,32,33], and the other focuses on the exposure/vulnerability of societies/spaces/economic systems to climate changes [34,35,36,37,38]. The functioning of mankind depends on both of these elements. However, vulnerability is essential from the humanist perspective as it takes human existence into consideration on many levels. According to FAO and IPCC [39,40,41,42], the VCC varies in time and space depending on multiple factors. A system’s vulnerability to climate change and variability depends on the nature, extent, and pace of the climate change and variability to which the system is exposed, its sensitivity, and adaptive capacity [39,40,41,42]. Exposure to climate change is thought to depend on location. For example, communities in semi-desert areas can be more vulnerable to droughts, whereas coastal areas can be more vulnerable to rising sea levels and cyclones. Sensitivity is the degree to which an organism is directly or indirectly affected by climate change and variability [41]. A tropical ecosystem will be less vulnerable to a decrease in rainfall than a semi-desert ecosystem because of successive water flows. Moreover, communities in mining areas will be less vulnerable to changing rainfall patterns than those dependent on agriculture relying on natural precipitation (rain) for irrigation [41,42,43,44]. Brooks et al. [45] note that the vulnerability depends on the context, and it can vary from one region to another. Therefore, understanding the weaknesses is a key step before taking a decision on orientation toward adaptive assistance [46,47]. Vulnerability and exposure are dynamic, they vary depending on the temporal and spatial scale, and they depend on economic, social, geographic, demographic, cultural, institutional, governmental, and environmental factors. Individuals and communities are exposed and vulnerable to threats to a different extent, and this is a consequence of such factors as wealth, education, race/ethnicity/religion, sex, age, class/caste, disability, and health status [48].

The diverse approaches to vulnerability to climate change have resulted in various indices embracing various attributes and territorial ranges [49,50,51]. Table 1 lists a group of the most popular indices published in the literature, with the basic determinants included in them and the spatial range for which it was developed.

Approaches to studying vulnerability are based on such disciplines as anthropology, geography, sociology, disaster management, climatology, and studies on sustainable sources of income [42,48]. Hahn et al. [43] suggested that vulnerability to climate change should be examined at a community level to understand and compare the community sensitivity within a town or region.

Exposure concerns the extent of change that took place in a meteorological aspect. It may include measures that indirectly describe the impact of meteorology on mankind and the environment. Many authors suggest that it can be measured with various indices [52,53,54] calculated on a local, regional or global scale [46]. However, the availability of high-quality data in an appropriate format is often limited [47,48], which, unfortunately, hinders comparative analyses on a global scale.

Sensitivity is the degree to which an element/economic system/species is likely to be affected by a change (or to respond to it). Sensitivity reflects a system’s exposure to climate impacts and is affected both by socio-economic and environmental conditions [34,55,56,57,58]. With the focus on a community living and working in rural areas, sensitivity includes human and environmental attributes which characterize the community in question and the space/economic system in which these people function (live and work).

Adaptive capacity in vulnerability studies is often referred to as “adaptability”, i.e., the ability of entities to adapt to new conditions in a system and affecting it. A society can affect adaptive capacity by building interactions between human and environmental elements of a system [59,60]. Therefore, the higher the adaptive capacity in a system, the higher the probability that the system will be resistant to climatic stress. The adaptive capacity is described in the literature on the subject as a feature with a transformative potential [61]. In vulnerability management, adaptive capacity affects modulation between maintaining a status quo and the system transformation to a new state, depending on which one is the most “desired” [62,63]. Higher adaptive capacity increases the probability of system maintenance [64,65,66,67,68,69,70,71]. The adaptive capacity of communities is determined by their socioeconomic characteristics [72,73,74,75,76,77,78,79,80,81,82,83,84].

Developing green energy affects the daily life of society living in rural areas [25,26,27,28,29]. It is also one of the targeted solutions, of key importance, in reducing greenhouse gas emissions into the atmosphere [3,5,20,29]. In recent years, government programs aimed at replacing non-renewable sources, with renewable energy sources have been spreading in many countries (including Poland) [85].

The goal of the study was to analyze socio-economic vulnerability to climate change in the context of green energy development. The study established the following research hypotheses: (1) socio-spatial vulnerability to climate change includes the three aspects of sensitivity, exposure, and adaptive capacity; (2) the availability of homogeneous indicators describing sensitivity, exposure, and adaptive capacity allows the estimation and comparison of vulnerability between different spatial units; (3) the development of green energy (acr. RES) may affect the reduction of vulnerability to climate change for the area under consideration. The study is a novel contribution to research on climate change vulnerability in rural areas, particularly in the aspect of attempts to demonstrate whether the rapid expansion of solar installations has been reflected in a reduction in vulnerability to climate change. The implementation of the main research objective was based on five detailed objectives: (1) analysis of available and homogeneous indicators describing the socioeconomic aspect of sensitivity, exposure, and adaptation of spatial units to climate change; (2) estimation of the VCC index using a general synthetic model; (3) development of three scenarios (VCC1, VCC2, VCC3) assuming, in part, the development of green energy production; (4) analysis of the temporal dynamics of variation in the main variables; and (5) investigating the spatial heterogeneity of the VCC scenarios.

## 2. Materials and Methods

**Figure 2:**
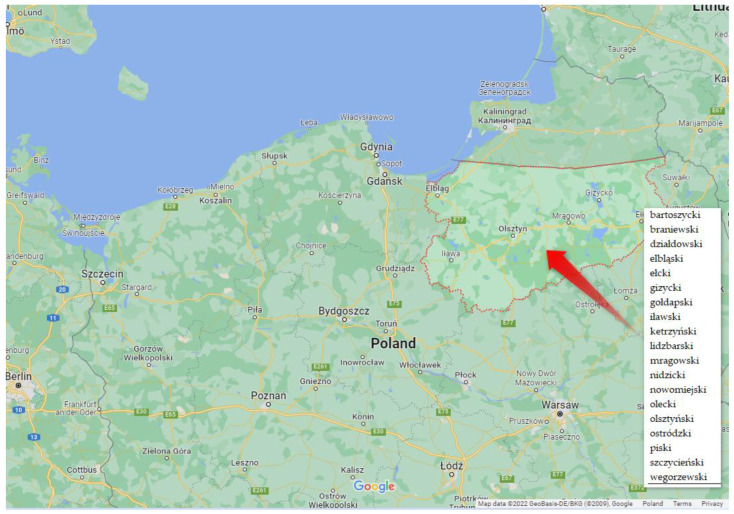
Location of the study area. Source: [86].

The comparative study covered the region of the Great Masurian Lakes. It is situated in the northeast of Poland, in the Warmińsko-Mazurskie Voivodship (see Figure 2). It occupies an area of 24,173.47 km^2^, and its population size is 1,436,367 people [64]. The region borders Russia in the north (the border is 208.3 km long). It is situated within the Central European Plain and the East-Baltic-Belorussian Plain. The land surface in the study area is of a lowland nature. The region’s climate is described as transitional, marine-continental. The weather is highly variable on a daily and annual scale. The characteristic features of the climate include rather cool summers and mild winters in the western part. The climate in the eastern part of the region is rather continental; summers are drier and hotter, and the winters are severe. The climate differences in the west and in the east of the region manifest themselves, e.g., in the average number of days with the snow cover—approx. 100 days in the east and 60 in the west. Characteristic natural features of the region include forests and lakes. The richness of the natural environment makes the air pollution level very low. Nearly the whole region is covered by a programme called “Zielone Płuca Polski” [The Green Lungs of Poland]. Agriculture is the leading branch of the economy. The agricultural yield is among the highest in Poland, and the average rye output per hectare amounts to 39.4 dt, with a national average of 35.9 dt.

The choice of this region as the study area is attributed to its agricultural nature and the presence of large natural areas, and the dependence of the main branch of the economy on weather conditions. Owing to governmental support, the regions have seen great interest in the installation of devices that acquire electricity from natural energy sources.

Human populations are vulnerable to the impacts of climate change largely because of the socioeconomic and political context in which they live. Thus, vulnerability to climate change is highly differentiated across geography, income levels, type of livelihood, and governance arrangements, among other things. Human vulnerability can be evaluated in terms of a range of different outcomes such as food security or household income. This study is focused on the socio-economic vulnerability to climate change index (VCC) defined as the degree to which a system (the population living in rural areas and engaged in agricultural production) is susceptible to and unable to cope with, adverse effects of climate change, including climate variability and extremes. This study considers three components (exposure, sensitivity, and adaptive capacity), expressed by indices that include the characteristics of the agricultural society, production, and weather extremes and the financial consequences they bring.

Generally, exposure is expressed as changes in average temperatures, changes in the annual precipitation, the frequency of extremely dry or wet months during the growing season, the weather anomaly index, and the flood threat index [67,68,69,70]. Exposure in the study covers three indices (X1–X3) (see Table 2). Unlike the weather indices determined for large areas, the indices adopted show the diversity of exposure to climate changes in micro-locations. Emphasis was placed on average financial losses in agricultural production caused by extreme weather phenomena and atmospheric anomalies. They were calculated on an annual scale for the area under study and expressed as pecuniary units.

Authors of many studies have expressed sensitivity as the area/share of soil used for agriculture, the number of farms whose activities include agricultural production, the rural area population, the area of agricultural land per person, and population density [2,87,88,89]. Sensitivity was expressed with ten indices (X4–X13) (see Table 2). They included mainly the attributes describing the society which inhabits rural areas and production in these areas. These are the components which are the most vulnerable to climatic changes as VCC covers the rural society and agriculture production is directly dependent on natural weather conditions.

The adaptive capacity usually covers unemployment rate, GDP per capita, alphabetization rate, percentage of the population with higher education, the number of schools per 1000 people, the number of physicians per 1000 people, the length of paved roads, social capital expressed as the membership of various social organizations or NGOs and the effectiveness of local management [87,88,89,90,91,92,93,94,95]. Adaptive capacity (X14-X38) includes 24 indices representing human and social capital, financial capital, and physical capital on rural areas (see Table 2). They cover the most important elements important from the adaptation perspective.

The selection of these indicators was based on the homogeneity of their occurrence for the studied spatial units. Poland is a unitary country with administrative uniformity. Units of administrative division are organized in the same manner and subordinated to the same extent to central bodies which determine their system and competence. It directly affects the sets of collected data as they are uniform in a given unit of administrative division. This makes it possible to compare vulnerability to climate change in specific time intervals.

Table 2 shows the parameters taken into account in the study of vulnerability to climate change and the basic descriptive parameters.

The vulnerability of exposed human and natural systems is a component of risk. Approaches to analyzing and assessing vulnerability have evolved. A general definition of vulnerability considers sensitivity, exposure, and adaptive capacity. However, it is not possible to calculate the level of VCC homogeneously for every country, region, natural system, or social group. The problem appears in the availability of uniform data and the significance of particular attributes for the issue under consideration. Vulnerability is widely understood to differ within communities and across societies, regions, and countries, as they are also changing through time [87].

In order to demonstrate the impact of green energy development on vulnerability to climate change (VCC), a general synthetic model was applied [96,97], which employs many components and takes into consideration their equivalence (1):

(1) $$VCC_{ik}=\frac{100}{n}\overset{n}{\underset{j=1}{\sum }}\alpha_{j}x_{ij}$$

Formulas (2) and (3) were used for the index normalization [96,97]:

(2) $$-\;\mathrm{for}\;\mathrm{stimulants}\;x_{ij}=\frac{x_{ij}-minx_{ij}}{\max x_{ij}-minx_{ij}}$$

(3) $$-\;\mathrm{for}\;\mathrm{destimulants}\;x_{ij}=\frac{maxx_{ij}-x_{ij}}{\max x_{ij}-minx_{ij}}$$

**Figure 3:**
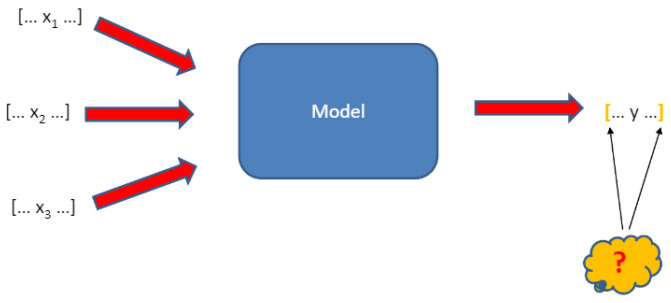
Model of sensitivity analysis.

The sensitivity analysis was also applied in the study [98] (see Figure 3), and it showed how a change in individual VCC elements, e.g., the size of new installations generating electricity from renewable sources, affects the vulnerability to climate change.

The sensitivity analysis forecasts the result with the use of variable systems (components) which affect them and provides a better understanding of the input and output variables [98]. The sensitivity analysis in this study took into consideration three scenarios. The first scenario (VCC1) included an assessment of the vulnerability index before implementation of the green energy support programs (base year—2017), the second scenario (VCC2)—an assessment of the vulnerability index following implementation of green energy support programs, with the other parameters unchanged, the third scenario (VCC3)—an assessment of vulnerability assuming a change in the production of green energy (X15), greenhouse gas emissions (X14), the amount of financial loss incurred (X1) because of extreme weather phenomena and weather anomalies and their number (X2) as well as electricity consumption (X38).

In order to assess the spatial variability of important indicators, it was determined to use the spatial autocorrelation test statistic [78]. It evaluates the correlation of a variable with respect to spatial location (a measure of the match between attribute similarity and location similarity). Global Moran’s index *(I)* indicates if a spatial correlation exists in the areas of the analyzed field [99,100,101,102]. Global Moran’s provides a way to determine the heterogeneity of the analyzed indicators using Formulas (4) and (5) [99,100,101,102]:

(4) $$I_{k}=\frac{n}{S_{0}}\frac{\sum_{i=1}^{n}\sum_{i^{\prime }=1,i^{\prime }\neq i}^{n}w_{ii^{\prime }}(x_{ij}-{\bar{x}}_{j})(x_{i^{\prime }j}-{\bar{x}}_{j})}{\sum_{i=1}^{n}{\left(x_{ij}-{\bar{x}}_{j}\right)}^{2}}$$

where

(5) $$S_{0k}=\sum_{i=1}^{n}\sum_{i^{\prime }=1}^{n}w_{ii^{\prime }}$$

Significance testing of the Moran’s *I* statistic is done using a test that verifies the following hypotheses:

**H0:**  *spatial autocorrelation does not exist;*

**H1:**  *spatial autocorrelation exists.*

(6) $$Z{\left(I\right)}_{k}=\frac{I_{k}-E\left(I_{k}\right)}{\sqrt{Var{\left(I\right)}_{k}}}$$

where

(7) $$E\left(I\right)=\frac{-1}{n-1}$$

(8) $$Var{\left(I\right)}_{k}=\frac{1}{w_{0}^{2}\left(n^{2}-1\right)}\left(n^{2}w_{1}-nw_{2}+3w_{0}^{2}\right)-E^{2}\left(I_{k}\right)$$

(9) $$w_{0}=\sum_{i=1}^{n}\sum_{j=1}^{n}w_{ij}$$

(10) $$w_{1}=0.5\sum_{i=1}^{n}\sum_{*i=1}^{n}{\left(w_{ii^{\prime }}+w_{i^{\prime }i}\right)}^{2}$$

(11) $$w_{2}=\sum_{i=1}^{n}{\left(w_{j*}+w_{*j}\right)}^{2}$$

**Symbol**

**Feature**


$VCC_{ik}$

index of vulnerability to climate change for *i*-th poviat in *k*-th scenario

$\propto_{j}$

value of weight of *j*-th variable

$x_{ij}$

value of *j*-th variable for *i*-th poviat

$max\;x_{ij}$

the maximum value of the *j*-th variable for *i*-th poviat

$min\;x_{ij}$

the minimum value of the *j*-th variable for *i*-th poviat
*I_k_*
the Global Moran’s index for *k*-th scenarionthe total number of poviats

$x_{ij,}\;x_{i^{\prime }j}$

the values of *j*-th variable for *i*-th and *i*’th compared poviats

${\bar{x}}_{j}$

mean value of the variable for all *j*-th poviats

$w_{ii^{\prime }}$

elements of the spatial matrix of weights for *i*-th and *i*’th poviat

$S_{0k}$

The value of aggregate weights in *k* scenario
*Z(I)_k_*
the number of standard deviations of Moran’s *I* in relation to the mean in kth scenario
*E(I)_k_*
the expected value of Moran’s *I* in kth scenario
*Var(I)_k_*
the variance value of Moran’s *I* in kth scenario

$w_{i^{\prime }i}$

elements of the spatial matrix of weights for *i’*th and *i*-th poviats

$w_{j*}$

quantity of sum of weights in the column of *j*-th variable

$w_{*j}$

quantity of sum of the weights in the row of *j*-th variable

The study used a threshold of 1.96 (calculated on the basis of (6)) in order to test the significance level of the *Z(I)* statistic. If the obtained value is greater than 1.96 or less than −1.96, it means that the spatial autocorrelation is significant [99,100,101,102]. The significance level of the study was *p* < 0.05.

## 3. Results

### 3.1. Changes in Green Energy (X15)

**Figure 4:**
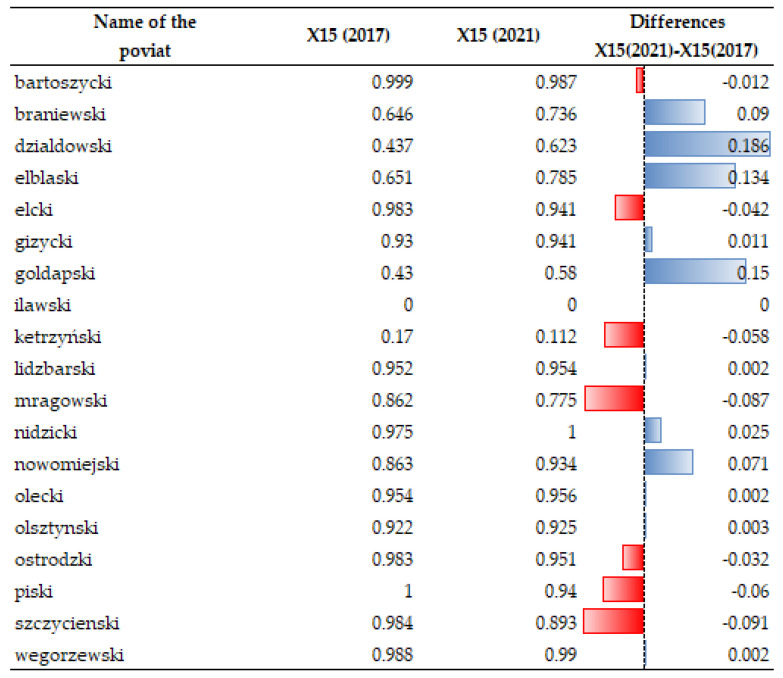
Changes in the index of green energy production (X15) in 2017–2021. Source: own study on [103].

**Figure 5:**
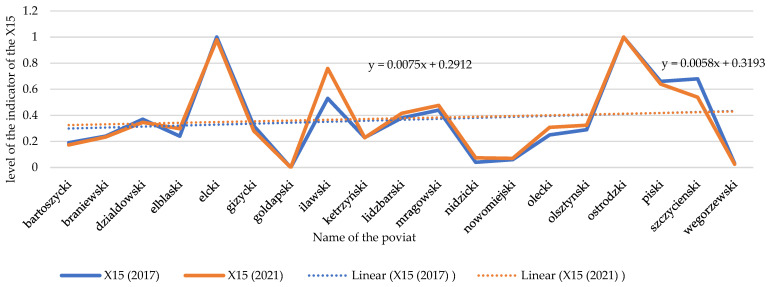
Temporal dynamics of green energy production (X15). Source: own study.

The level of vulnerability to climate change provides important guidance to local stakeholders concerning investment and climate policies. Legislation changes and implementation of government programs aimed at providing financial support for the development of installations for electricity generation from renewable sources slow down climate change. Many programs supporting a reduction of heat and energy loss and utilization of natural renewable sources have been implemented in Poland. For example, the “Mój prąd [My electricity]” program provides financial support to those who want to invest in electricity generation from renewable energy sources. Owing to this measure, people eagerly invested their money in such energy sources, which resulted in an increase in the number and power of installed photovoltaic, wind, hydro, geothermal, biogas and biomass systems. Temporal dynamics of X15 for data 2017 and 2021 showed that the greatest difference in the generation of such energy from renewable sources in the area under study was noted in the counties of Braniewski, Działdowski, Elbląski, Gołdapski, and Nowomiejski (see Figure 4 and Figure 5).

### 3.2. Changes in Greenhouse Gas Emissions (X14)

**Figure 6:**
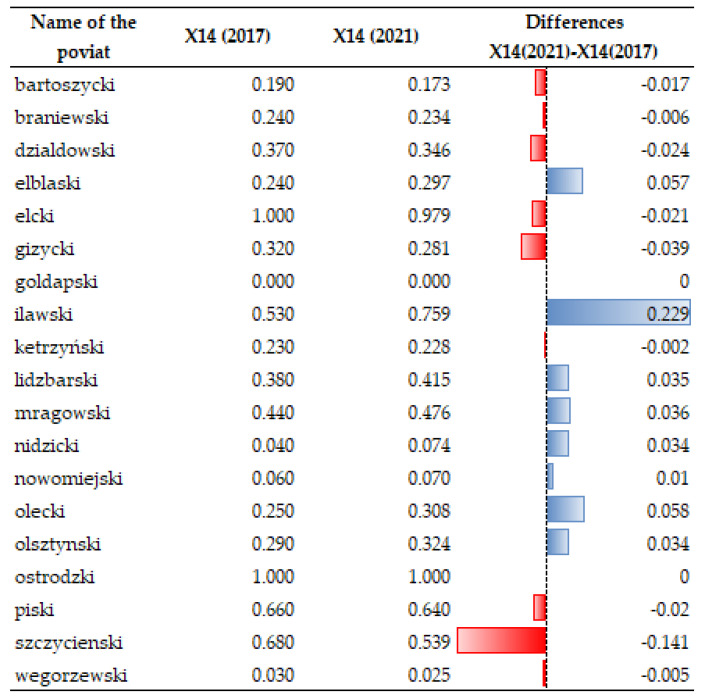
Changes in the index of greenhouse gas emissions (X14) in individual spatial units (tons/per year). Source: own study on [104].

**Figure 7:**
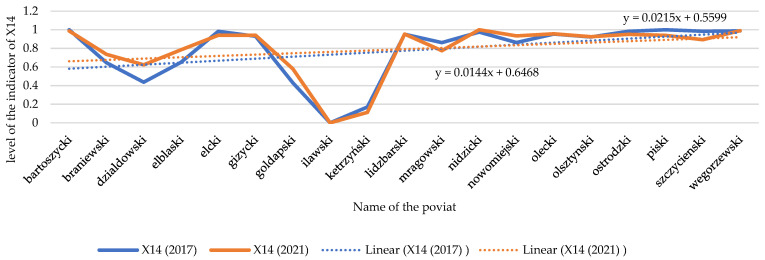
Temporal dynamics of greenhouse gas emissions (X14). Source: own study.

Growth in installations used to generate electricity from renewable sources does not always go hand-in-hand with a reduction of greenhouse gas emissions. Analyzing temporal dynamics of X14, a decrease in greenhouse gas emissions is noticeable in nearly all the analyzed units during the time interval under study, but there are some in which the emission increased. This especially applies to the counties of Elbąski, Iławski, Nidzicki, Nowomiejski, Olsztyński, and Olecki (see Figure 6 and Figure 7).

### 3.3. Changes in Electricity Consumption (X38)

**Figure 8:**
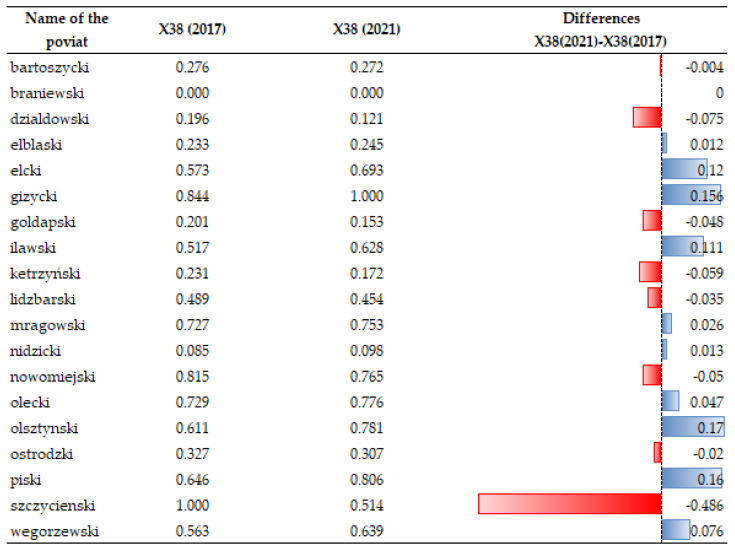
Changes in the index of the electricity consumption/ per person (X38) in individual spatial units. Source: own study on [106].

**Figure 9:**
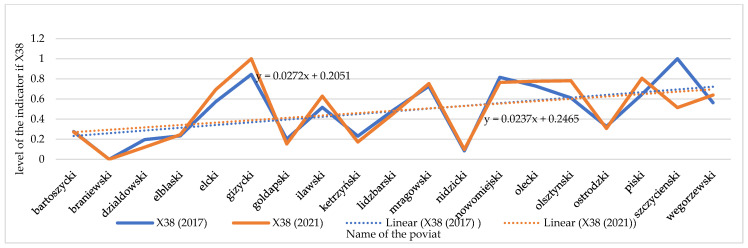
Temporal dynamics of the electricity consumption/ per person (X38). Source: own study.

Changes in electricity consumption per person have been emphasized in the analyses (X38). As the data analysis showed (see Figure 8 and Figure 9), the temporal dynamics of X38 have been increasing in recent years despite its high price and promotion of “energy saving”. Surprisingly, there are seven units where electricity consumption was found to have decreased: in the counties of Działdowski, Gołdapski, Kętrzyński, Lidzbarski, Nowomiejski, Ostródzki and Szczycieński. Local authorities in those units implemented “low-emission policy” measures, which resulted in, for example, the development of low-emission urban and agglomeration transport in the major urban centers of the region, the improvement of thermal insulation of public facilities, the replacement of windows, external doors, the installation of energy-saving lighting, the transformation of heating systems (including heat source replacement and connection), the installation of ventilation and air conditioning systems with the use of weather-dependent automation and building management systems, the installation of chimney filters, the construction/modernization of internal receiving installations and elimination of existing high-emission heat sources, and the transformation of production lines into more energy efficient lines [105]. Such measures taken by local authorities were intended to reduce greenhouse gas emissions to the atmosphere, and this effect is noticeable.

### 3.4. Changes in the Number of Extreme Events and Financial Losses in Farmland (X1, X2)

**Figure 10:**
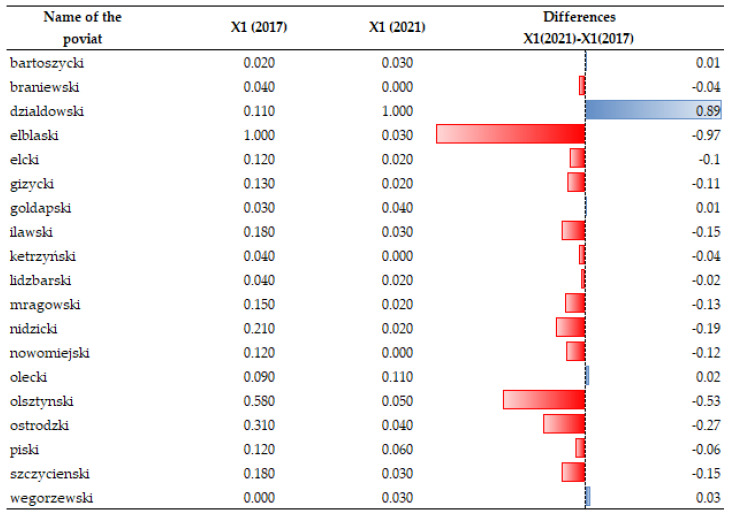
Changes in index of the financial loss (X1) caused by extreme weather phenomena and weather anomalies in individual spatial units. Source: own study on [108].

**Figure 11:**
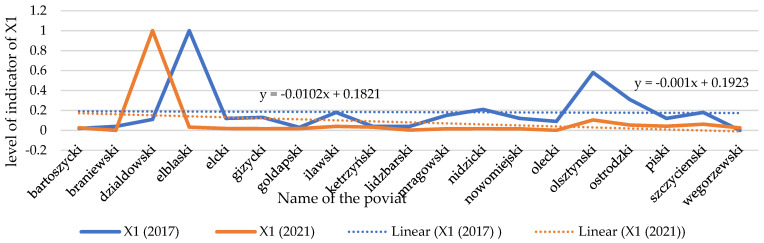
Temporal dynamics of the financial loss (X1). Source: own study.

**Figure 12:**
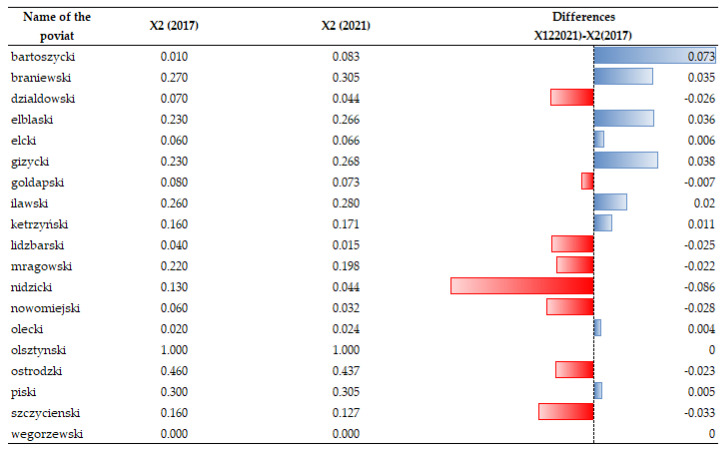
Changes in the index of the number of extreme weather phenomena and weather anomalies in the spatial units under study (X2). Source: own study.

**Figure 13:**
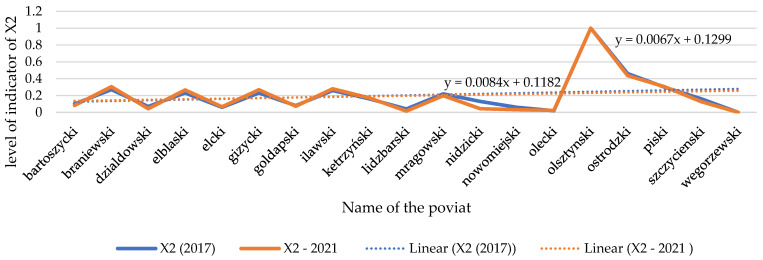
Temporal dynamics of the number of extreme weather phenomena and weather anomalies (X2). Source: own study.

A decrease in the indicator of financial loss (X1) is noticeable nearly in all spatial units under study (see Figure 10 and Figure 11). They are random in character, and they do not depend on the activities of the community in the region, although it affects the VCC level. The largest increase is noticeable in the county of Działdowski. Unfortunately, despite a decrease in the extreme phenomena in the area (see Figure 12 and Figure 13), the scale of their impact on rural agricultural land increased considerably. They were caused by hail, drought, torrential rain, and the negative effects of plant wintering as a result of frost or their overwatering between December and April [107]. That trend should be explained by the scale of the extreme events occurring in the Działdowski poviat and the unit value of agricultural crops, which are used to estimate financial losses. When assessing the scale of financial losses in agricultural crops, the average productivity of the soil and the market price of the crop are considered.

### 3.5. VCC Scenarios

**Figure 14:**
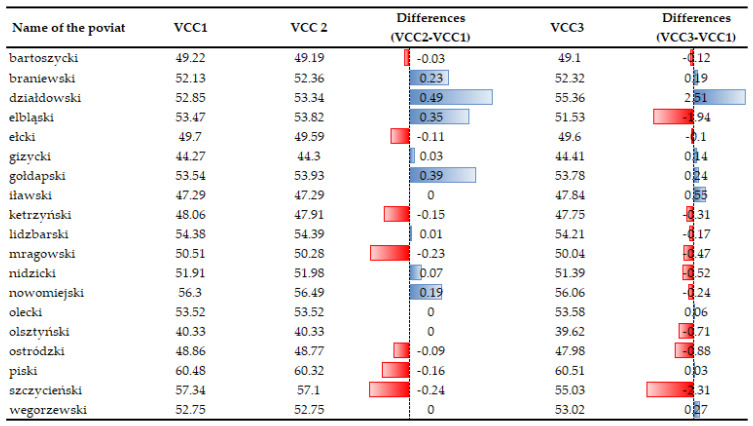
Sensitivity analysis for vulnerability to climate change (VCC1, VCC2, VCC3) taking into account various solution variants. Source: own study.

All the developed VCC1, VCC2, and VCC3 scenarios were analyzed on a regional scale. The results show that despite an increase in electricity production from renewable energy sources, there was no considerable decrease in the vulnerability to climate change. This is a consequence, *inter alia*, of the index structure and the normalization process, whose function is to minimize rapid changes in the index under study [96]. The VCC decreased in seven objects, it increased in eight and remained unchanged in four. Evidently, an increase in energy generation from renewable sources does not guarantee a decrease in the VCC (see Figure 14). This can be achieved only as a result of measures on a global scale. An analysis of the change of indices taken for the study (electricity generated from renewable sources—X15, greenhouse gas emission—X14, electricity consumption per person—X38, the number of extreme weather phenomena and weather anomalies and the amount of financial loss caused by them—X1, X2) shows a VCC decrease in 11 cases and an increase in seven. The largest vulnerability increase was noted in the counties of Działdowski, Iławski and the greatest decrease was in the counties of Elbląski, Szczycieński, Olsztyński, Ostródzki, Mragowski and Nidzicki.

A more detailed analysis of two cases: the largest VCC decrease (the counties of Szczycieński and Elbląski) and its largest increase (the county of Działdowski).

In the Szczycieński Poviat in 2021, the most important indicators studied (X1, X2, X14, X15, X38) decreased in value compared to 2017, which influenced the VCC. In Elbląski Powiat, only indicator X1 decreased, while the others increased. In the case of the Działdowski Powiat, the increase in vulnerability to climate change (VCC) in 2021 is remarkable. The X1 indicator—financial losses due to extreme events and weather anomalies—is primarily responsible for the increase in VCC.

In general, the effects of extreme weather events can be classified into direct and indirect effects. Direct concerns losses in agricultural crops and in the morale and health of agricultural producers. Indirect effects concern future economic performance, production of goods and services, and disruption of other sectors having a direct impact on the agricultural enterprise. Observable over a longer period of time after an extreme event [89]. A 2008 study by the Heliview Group [109] found that the main risks associated with weather disasters are increased costs (54%), reduced turnover (43%) and reduced profits (19%). Norrington and Underwood [110] highlighted risks in the form of livestock damage, which can affect subsequent farming operations. In addition, the occurrence of extreme weather events creates other costs relating to machinery replacement, among others [111]. Insurance rates are also rising significantly, which can be a problem for agricultural producers.

### 3.6. Spatial Heterogeneity of VCC1, VCC2, VCC3 and X1, X2, X14, X15, X38

Global Moran’s index *I* was calculated using licensed ArcGIS Pro software (ArcGIS_Desktop_107_167519). The spatial distribution of VCC scenarios characterizing each powiat was verified based on contiguity edges corners (see Table 3). The results are shown in Figure 12.

**Figure 15:**
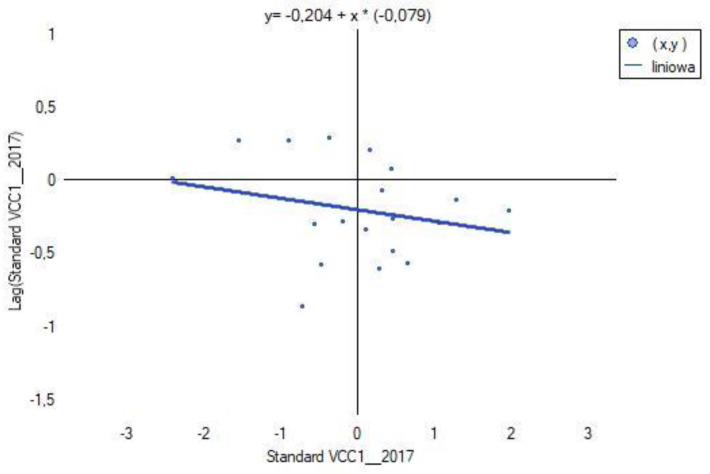
Chart of the global Moran’s index I for VCC1. Source: own study.

**Figure 16:**
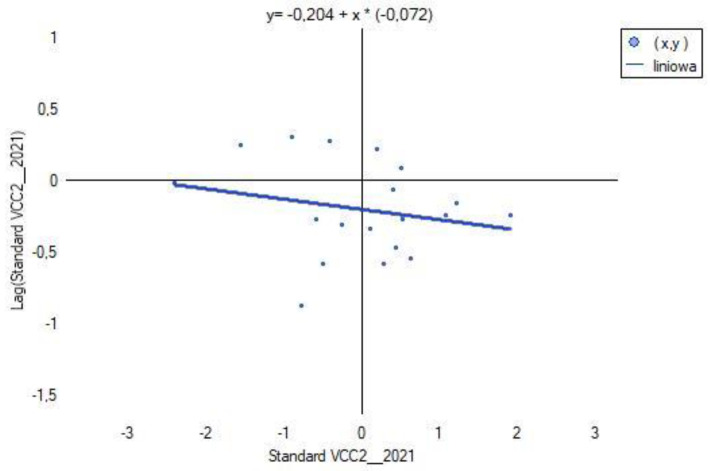
Chart of the global Moran’s index I for VCC2. Source: own study.

**Figure 17:**
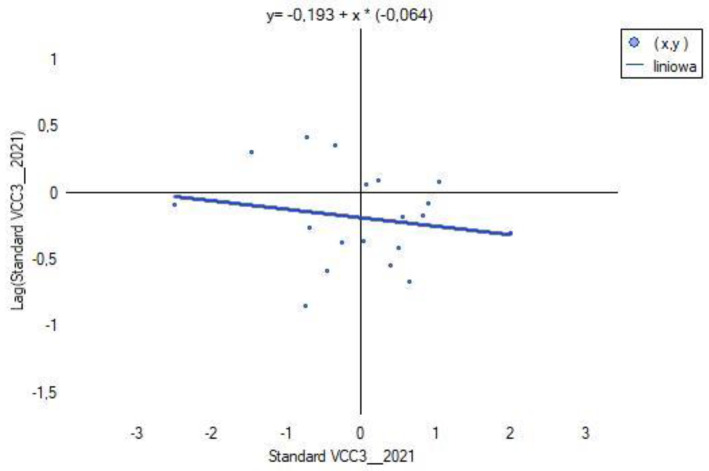
Chart of the global Moran’s index I for VCC3. Source: own study.

The results of computing for each of the VCC scenarios (see Figure 12) show a negative value of Moran’s index *I* and close to 0. This indicates that in the analyzed scenario, regardless of the fact that there have been changes in the indices X1, X1, X14, X15, X38, the spatial effect of agglomeration does not occur. The studied spatial units (powiats) do not merge into clusters, but occur as so-called hot spots (see Figure 15, Figure 16 and Figure 17). The test statistic for the significance of Moran’s autocorrelation coefficient (z-score) and the *p*-value pt level confirm that the null hypothesis of a random distribution of vulnerability to climate change (VCC) should be accepted.

The spatial distribution of the main indicators studied (X1, X2, X14, X15, X38) was also analyzed. Additionally, these variables also do not exhibit features tending toward agglomeration. They are characterized by a random spatial distribution for both the baseline (2017) and final year of analysis (2021) (see Table 4).

## 4. Discussion

Experts emphasized that although climate changes can be a consequence of natural processes on the Earth, recent human activities have accelerated the process. Therefore, it is necessary to implement some mitigating and adaptive measures as well, with respect to climate change. Humanity prospers in various climate zones, and the geographic location of countries/regions with a concentration of socio-economic activity in the climate change impact zones (deltas of large rivers, coastal strips) makes them more vulnerable to the effects of such changes. What might vulnerable societies which are partially or wholly dependent on natural resources (weather/soil/water) for their livelihoods do in response to substantially increased climate variability? Options include increasing the productivity of crop and livestock systems, diversifying farm income [112], and most importantly, increasing the capacity to adapt to climate change, mitigate potential risks, and manage the consequences. Adaptation to climate change is crucial. Replacing non-reproducible energy sources with reproducible sources [113,114,115] (from the sun/wind) is on the mandatory list of measures because these sources avoid emitting carbon dioxide and other greenhouse gases that contribute to global warming. A local increase in green energy is not enough to improve the resilience of spatial units to climate change. The results of the presented studies support the findings. The level of index of vulnerability to climate change (VCC1) from the reference year (2017) relative to VCC2 (2021) has changed slightly, despite the development of RES. The variation does not exceed a 1% reduction in the value of the VCC1 index. In contrast, the value of the VCC3 index, reflecting and including changes in green energy production (X15), electricity consumption/inhabitant (X38) and greenhouse gas emissions (X14), exhibited more favorably the impact of these indicators on vulnerability to climate change, compared to the VCC1 reference value. In eleven powiats, the VCC3 index decreased between 1 and 4%. In seven of these powiats, green energy production increased, resulting in an average 10% decrease in the X15 indicator, the X14 indicator representing greenhouse gas emissions decreased by an average of 7%, while the X38 indicator describing electricity consumption/per capita decreased by an average of 16%.

What implications arise from the research? Primarily, society in general has to act comprehensively and in multiple ways. The activities as a whole have to be focused on using energy in an economical manner, as it is the largest source of human-caused greenhouse gas emissions. The method by personal consumption and generation of energy greatly affects the climate. The sectors particularly affected are transportation, households, agriculture, and recycling. As a percentage of the EU’s total greenhouse gas emissions, these sectors are responsible for about 55% [116]. Emissions may be reduced by using cleaner energy sources, for example, replacing fossil fuels with non-fossil fuel renewable energy sources, reducing overall energy consumption levels, introducing measures to encourage energy conservation and attempting to improve energy efficiency, for example, by improving insulation in buildings and utilizing cleaner modes of transportation. Emission reduction efforts are supported by a spectrum of policy documents, long-term strategies and implemented programs including “My Electricity” presented in the case study. Considering the urgency of action, a question to be answered as well is whether there should be continued investment in fossil fuel-based energy? Political decisions to subsidize a particular energy source influence investment decisions. The implemented “My Energy” program was instrumental in increasing the amount of energy produced from renewable sources (solar/wind). Created conditions not only favorable for investors, but also demonstrated, the possibility of widespread action towards climate protection. Actions to achieve are creating a domino effect. The European Environment Agency (EEA) and the European Environment Information and Observation Network (EIONET) spotlighted the other innovative solutions used in many sectors as well, capable of reducing energy-related greenhouse gas emissions. Actions for example reducing food waste, urban gardening, streamlining supply chains and using solar energy in the airport transportation sector appear to be small elements of a large puzzle [116], but viewed together they show how innovative technologies and practices can pave the way for wider changes to ensure sustainability.

### Limitations of the Research

The construction of the indices used in this report was based on data obtained from third parties (statistical data, lists based on the collected data). Their quality cannot be guaranteed, although the data were acquired from renowned sources. The data sets obtained were subjected to detailed scrutiny and assessed for any inconsistency. The possibility of achieving comparability in as many regions as possible was the main factor taken into consideration when choosing primary data sources. The estimated and analyzed vulnerability to climate change index does not differentiate the forecast index increases and decreases relative to the base climate parameters, but rather it measures the degree to which a (human or natural) system will cope with a potential change in the present status. Indices for climate-related extreme events provide a general risk review for a location and show that exposure at individual sites in a region can vary.

The adaptive capacity can be examined on the country, local, community or individual level, the indices reflecting the adaptive capacity focus on the structural factors on the macro level, such as management and the economy. Its aim is to show the individual trend for vulnerability at a specific location.

The study assumes that all dimensions of vulnerability and the indicators are equally important. Future research by the study’s authors will assume a differential impact of individual indicators on the vulnerability to climate change index.

## 5. Conclusions

This study focuses on the impact of an increase in energy generation from renewable sources and the vulnerability to climate change. Construction of the VCC index was based on a model recommended by IPCC and FAO, taking into account the exposure, sensitivity, and adaptive capacity of spatial units/society. This index plays a very important role as it can provide guidance for decision-makers concerning adaptive and mitigating measures. The goal of the study was to test the impact of green energy development on vulnerability to climate change. The motivation for the study attempted was the development of green energy, conducted in recent years in Poland with the help of national funding. Studies demonstrated that taking single actions, for example, increasing green energy production, does not affect VCC levels significantly.

It is necessary to act comprehensively. Currently, the authorities of the spatial units studied are acting locally, without a coherent regional strategy. A confirmation is provided by the analysis of the spatial heterogeneity of VCC and the main variables. The results of the conducted research can guide regional and national authorities to take targeted actions involving the entire community. Only coordinated action can help achieve the effect of increasing resilience to climate change.

## Figures and Tables

**Table 1:** A list of indices describing vulnerability with their spatial range.

Acronym	Name	Description	Range (Level)
PVCCI	Physical Vulnerability to Climate Change	climatic shocks and countries’ exposure to these shocks	global, national
HCCVI	Habitat Climate Change Vulnerability Index	Exposure, resilience	regional, local
CVI	Climate Vulnerability Index	Exposure, Sensitivity and Adaptive Capability	regional, local
CHRI	Cultural Heritage Risk Index	hazard, exposure, vulnerability	regional, local
VI	Vulnerability index	a measure of the exposure of a population to some hazard	regional, local
EVI	Environmental Vulnerability Index	focus on planned solutions to negative pressures on the environment while promoting sustainability	national, regional
CVM	Climate Vulnerability Monitor	global assessment of the effect of climate change on the world’s populations brought together by panels of key international authorities	global, national
CCVI	Climate Change Vulnerability Index	enables organisations to identify areas of risk within their operations, supply chains and investments.	global, national
SeVI	Socioeconomic Vulnerability Index	climate change affected communities in seven unions	regional, local
SVI	Social Vulnerability Index	social vulnerability is a term describing how resilient a community is when confronted by external stresses on human health	regional, local
GEVI	Global Energy Vulnerability Index	exposure of the energy system to adverse events and changes	global, national
HVI	heat vulnerability index	vulnerability to extreme heat	local
LVI	livelihood vulnerability index	used to assess the vulnerability of farming households to climate change and variability	Local, regional
IVI	Infrastructure Vulnerability Index	Infrastructure Vulnerability Index for the drinking water distribution system to assess the vulnerability of the system to terrorist acts and to support the selection of investments to enhance security	Local, regional
HVI	health vulnerability index	integrated health vulnerability index	Local, regional
SDVI	Standardized drought Vulnerability index	vulnerability to drought and desertification	Local, regional
BCVI	Bat cave vulnerability index	a vulnerability index for bat species to climate change	Local, regional

Source: own study.

**Table 2:** A list of indices was taken into account in the VCC study with the main descriptive parameters.

Symbol	Features	Measure	Average	Min.	Max.	Variation Coefficient
**Exposure**						
X1	Financial loss on agricultural production	PLN/year/ha AL	32.0	11.2	57.0	39.75
X2	Extreme events	Index/ha AL	1.0	0.7	2.0	32.26
X3	Agroclimatic conditions	Index (IUNG)	8.0	5.2	10.0	13.31
**Sensitivity**						
X4	RPP	Index (IUNG)	65.0	52.2	78.0	12.15
X5	agricultural land	% of poviat	56	30	72	20.10
X6	rural population	% of poviat	51	32.6	75	22.08
X7	Number of individual farms	in poviat	2	1.2	4	31.71
X8	Farm area	ha/in poviat	55,561	30,153.9	93,758	30.22
X9	Income per farm	PLN/in poviat	2639	2427.8	29.1	4.74
X10	Employment in agriculture	%/in poviat	29	16.1	41	24.73
X11	Population density	People/km^2^	48	32	81	29.24
X12	Irrigation—drainage ditch area	ha AL	1791	318	5889	68.68
X13	Soil degradation (acidification)	%	43	19	72	36.63
**Adaptive Capacity**						
X14	Greenhouse gas emission	Tons/year	49,346	55,286	134,717	39,819.3
X15	RES	MW	19	0	86	135.57
X16	GDP	PLN	27,747	21,623.2	35,977	12.09
X17	Unemployment rate	%	14	4.8	22	32.08
X18	Number of schools	index	42	17	92	41.36
X19	Education (percentage of individuals with university education)	%	12	9	15	13.31
X20	Healthcare	Index/10,000 people	32	7.8	53	35.46
X21	Medical care	Index/10,000 people	402	173	930	46.19
X22	Density of paved roads	km/km^2^	117	66.9	182	25.07
X23	Urbanization rate	%	50	25.6	68	22.83
X24	Entities of national economy	Per 10,000 people	782	635	987	11.74
X25	Community initiatives	index	0	0.1	1	39.24
X26	Main crop yield	dt/ha	37	29.5	47	11.01
X27	Area under crops	ha	1933	1083	3516	32.25
X28	Local government management level	PLN/resident	25,907	12,510	56,495	49.68
X29	Human capital	index	27	3	51	48.11
X30	Economic migration	Number of individuals	952	378	2071	43.81
X31	Economic capital	PLN	1,296,471	126,373	3,029,313	73.05
X32	Number of families with children	index	22,207	7859	44,120	43.88
X33	Fertility rate	index	1	1.1	2	8.20
X34	Water supply infrastructure	km	828	294.9	1910	44.65
X35	Natural resources	%	47	19	78	34.38
X36	Housing resources	number	20,711	8462	43,168	45.72
X37	Sewerage infrastructure	km	357	141.7	1037	66.85
X38	Energy consumption	GWh	44	18.1	99	47.20

AL—area of agriculture land; IUNG—Instytut Upraw, Nawożenia i Gleboznastwa (the national research unit that developed the index; PLN—currency in use in Poland; MW—megawatt; dt—deciton; GWh—gigawatt hour. Source: own study.

**Table 3:** Global Moran’s *I* for VCC.

Indexes	VCC1 (2017)	VCC2 (2021)	VCC3 (2021)
Moran’s I Index	−0.07882	−0.07233	−0.06382
Expected Index	−0.05555	−0.05556	−0.05556
Variance	0.02000	0.02017	0.01970
z-score	−0.16451	−0.11815	−0.05887
*p*-value	0.86933	0.90595	0.95305

**Table 4:** Global Moran’s *I* for X1, X2, X14, X15, X38.

Indexes	X1_2017	X1_2021	X2_2017	X2_2021	X14_2017	X14_2021	X15_2017	X15_2021	X38_2017	X38_2021
Moran’s Index	−0.030147	−0.07041	−0.04269	−0.10904	−0.09442	−0.15542	0.073736	0.102049	0.129615	0.15554
Expected Index	−0.055556	−0.05556	−0.05556	−0.05556	−0.05556	−0.05556	−0.05556	−0.05556	−0.05556	−0.05556
Variance	0.012367	0.000958	0.01142	0.012547	0.019677	0.017273	0.020595	0.020891	0.022142	0.022599
z-score	0.228482	−0.48001	0.120434	−0.47749	−0.27706	−0.75988	0.900932	1.090402	1.244398	1.404233
*p*-value	0.819272	0.631219	0.904139	0.633017	0.781737	0.447329	0.367624	0.275536	0.213353	0.16025

## Data Availability

Not applicable.
